# GeneChip Expression Profiling Reveals the Alterations of Energy Metabolism Related Genes in Osteocytes under Large Gradient High Magnetic Fields

**DOI:** 10.1371/journal.pone.0116359

**Published:** 2015-01-30

**Authors:** Yang Wang, Zhi-Hao Chen, Chun Yin, Jian-Hua Ma, Di-Jie Li, Fan Zhao, Yu-Long Sun, Li-Fang Hu, Peng Shang, Ai-Rong Qian

**Affiliations:** Key Laboratory for Space Biosciences & Biotechnology, Institute of Special Environmental Biophysics, School of Life Sciences, Northwestern Polytechnical University, Xi’an 710072, China; Temple University School of Medicine, UNITED STATES

## Abstract

The diamagnetic levitation as a novel ground-based model for simulating a reduced gravity environment has recently been applied in life science research. In this study a specially designed superconducting magnet with a large gradient high magnetic field (LG-HMF), which can provide three apparent gravity levels (μ-g, 1-g, and 2-g), was used to simulate a space-like gravity environment. Osteocyte, as the most important mechanosensor in bone, takes a pivotal position in mediating the mechano-induced bone remodeling. In this study, the effects of LG-HMF on gene expression profiling of osteocyte-like cell line MLO-Y4 were investigated by Affymetrix DNA microarray. LG-HMF affected osteocyte gene expression profiling. Differentially expressed genes (DEGs) and data mining were further analyzed by using bioinfomatic tools, such as DAVID, iReport. 12 energy metabolism related genes (*PFKL, AK4, ALDOC, COX7A1, STC1, ADM, CA9, CA12, P4HA1, APLN, GPR35* and *GPR84*) were further confirmed by real-time PCR. An integrated gene interaction network of 12 DEGs was constructed. Bio-data mining showed that genes involved in glucose metabolic process and apoptosis changed notablly. Our results demostrated that LG-HMF affected the expression of energy metabolism related genes in osteocyte. The identification of sensitive genes to special environments may provide some potential targets for preventing and treating bone loss or osteoporosis.

## Introduction

High magnetic fields (HMFs) are one of the most powerful tools for studying the properties of materials because they couple directly to the electronic charge and magnetic moments of the protons, neutrons, and electrons [1]. Recent technologic innovations have led to the generation of man-made static magnetic fields up to 10 Tesla (T). HMFs produced by a superconducting magnet have been widely used in research and medical applications. HMFs (>10 T) affected the cell cytoskeleton, cell viability and differentiation [2], significantly retarded *Xenopus laevis* development and suppressed gene expression [3].

Recently, scientists in several national HMF laboratories, including Japan [3], Nijmegen [4], the USA [5] and France [6] have been carried out studies in physics, chemistry, materials, and biology using a large-gradient, high-magnetic field (LG-HMF) environment. The magnetic body force (Kelvin force), like gravity, is a body force and the counterbalance between the magnetic force and gravity holds for each molecule constituting the materials [7]. If the magnetic field is strong enough, magnetism can affect any atom or molecule. In addition, the LG-HMF imposes a directional ponderomotive force on diamagnetic substances, and thus can simulate gravity or accelerative forces with the advantage that it can be confined to small areas [8, 9]. Therefore, the magnetic body forces produced by LG-HMF can be used to simulate different gravity environments, which is one of the most promising tools to realize a virtual microgravity environment on earth. Impressive records of levitating insects, strawberries, frogs, mouse, water drop, plants, and mammalian cells have been reported [4, 5, 10–17]. A diamagnetic levitation technical platform has been developed by our laboratory, and we have successfully carried out experimental research, including cell culture, embryogenesis of model animals, protein crystallization, and microbiology [18]. Our findings showed that diamagnetic levitation using superconducting magnet affects the morphology, cytoskeleton architecture, and function of bone cells (osteocytes, osteoblasts and osteoclasts) [19–27] and the development of silkworm eggs [28].

Osteocyes are terminally differentiated from osteoblasts and play a crucial role in bone remodeling [29]. Osteocytes take up more than 90% of all bone cells [30, 31]. In mature bone, osteocytes are embed in the mineralized matrix and these dendritic cells connecte with each other and to the bone surface through the lacuno-canalicular system[32]. The location, mophology and network of osteocyte made it as an ideal candidate for systemic homeostasis regulation. Osteocytes may paly a pivotal role in mediating the function of osteoblast and osteoclast[33, 34]. The apoptosis of osteocyte has been proved to be crucial in stress/unstress induced bone remodeling through regulating bone formation and resorption processes[35, 36]. More and more studies have been reported that osteocytes in bone tissue are very sensitive to mechanical stimulus and maybe are one of the most important mechanosensors [37, 38].Our previous studies have reported that diamagnetic levitation causes changes in the morphology, cytoskeleton, and focal adhesion proteins expression in osteocytes [23].

Although some studies on the biological effects of diamagnetic levitation have been carried out, reports on the effects of diamagnetic levitation on mammalian cells are still limited. In addition, there are also a few reports on the effects of weightlessness on osteocytes’ structure and function. The purpose of this study is to further explore the possible mechanism of cellular morphology and function alterations induced by LG-HMF. The identification of specific mechanosensitive genes will improve our understandings of physiological effects observed during spaceflight and may provide some new clues to further investigate the mechanism of bone loss induced by weightlessness. Morevoer, findings at a cellular level may provide some evidences for the application of superconducting magnet into biological research.

## Results

### Effects of LG-HMF on gene expression profiles of MLO-Y4 cell line

**Figure 1:**
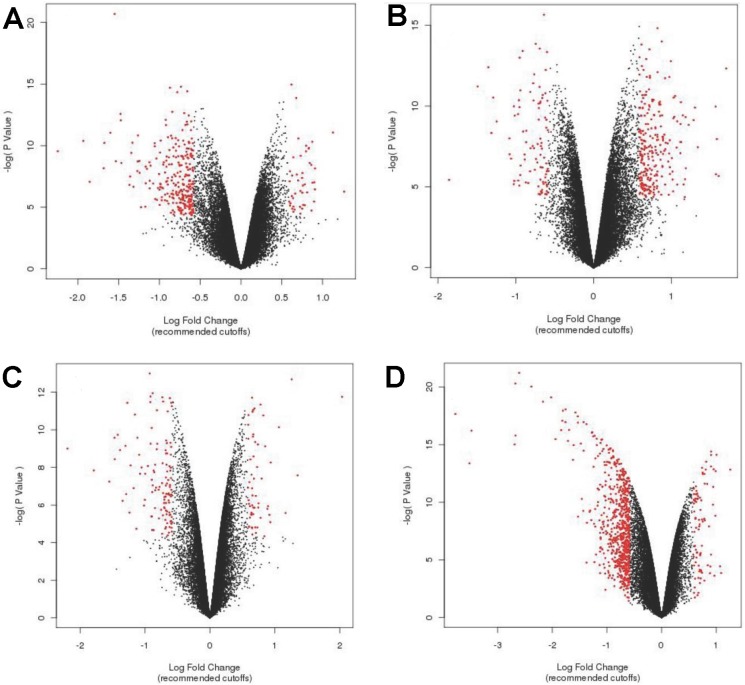
Volcano plots of differentially expressed genes in MLO-Y4 cells exposed to LG-HMF. Volcano plots displays unstandardized signal against noise-adjusted/standardized signal. The x-axis represents the fold change cutoff, while y-axis shows the negative logarithmic of *P* value. A: μ-g *v.s.* control (set 1), B: 2-g *v.s.* control (set 2), C: 1-g *v.s.* control (set 3), D: μ-g *v.s.* 2-g (set 4).

In this study, a special designed superconducting magnet with large gradient high magnetic field was used to simulate different gravity levels. For the convenience of description, we named four sets as set 1(μ-g *v.s.* control), set 2 (2-g *v.s.* control), set 3 (1-g *v.s.* control) and set 4 (μ-g *v.s*. 2-g).The volcano plots in Fig. 1 showed the overall feature of the four gene sets in MLO-Y4 cells exposed to LG-HMF. Down-regulated genes were much more than up-regulated genes in set 1(Fig. 1A), while it presented a reversed feature in set 2 (Fig. 1B). Set 3 contained the least number of genes with lower fold change than the other three sets (Fig. 1C). In set 4, there was a significant increase in down-expressed genes compared to other sets (Fig. 1D).

The DEGs were refiltered by setting the cutoff limitations. Besides the initial fold change, cutoff of 2- and 1.5- fold were applied. The number of DEGs (FC > 1.5 and 2) in 4 sets were shown in Table 1. The number of genes decreased with the increase of the fold change. There are a few more down-regulated genes in the set1, set 3 and set 4 relative to set 2, but in set 2 there are much more up-regulated genes compared with other sets (Table 1). In set 1 and set 3, all DGEs (FC > 2) were down-regulated genes. In set 2, the number of up-regulated DGEs was more than that in down-regulated DGEs.

**Table 1:** Number of DEGs in MLO-Y4 cells exposed to LG-HMF.

**group**	**FC (2)**		**FC (1.5)**	
	**upregulated**	**downregulated**	**Upregulated**	**downregulated**
Set1(μ-g *v.s.* Control)	0	14	7	40
Set2(2-g *v.s.* Control)	13	5	69	40
Set3(1-g *v.s.* Control)	0	13	4	34
Set4(μ-g *v.s.* 2-g)	2	40	31	137

DEGs: differentially expressed genes. FC: Fold change. This table listed the number of genes up-expressed or down-expressed with cutoff limitations of 2-fold and 1.5-fold. Those genes were the DEGs obtained from the comparison groups between three experimental treatments (μ-g, 1-g and 2-g) and control, also with the group of μ-g *vs*. 2g (*P* <0.05).

**Figure 2:**
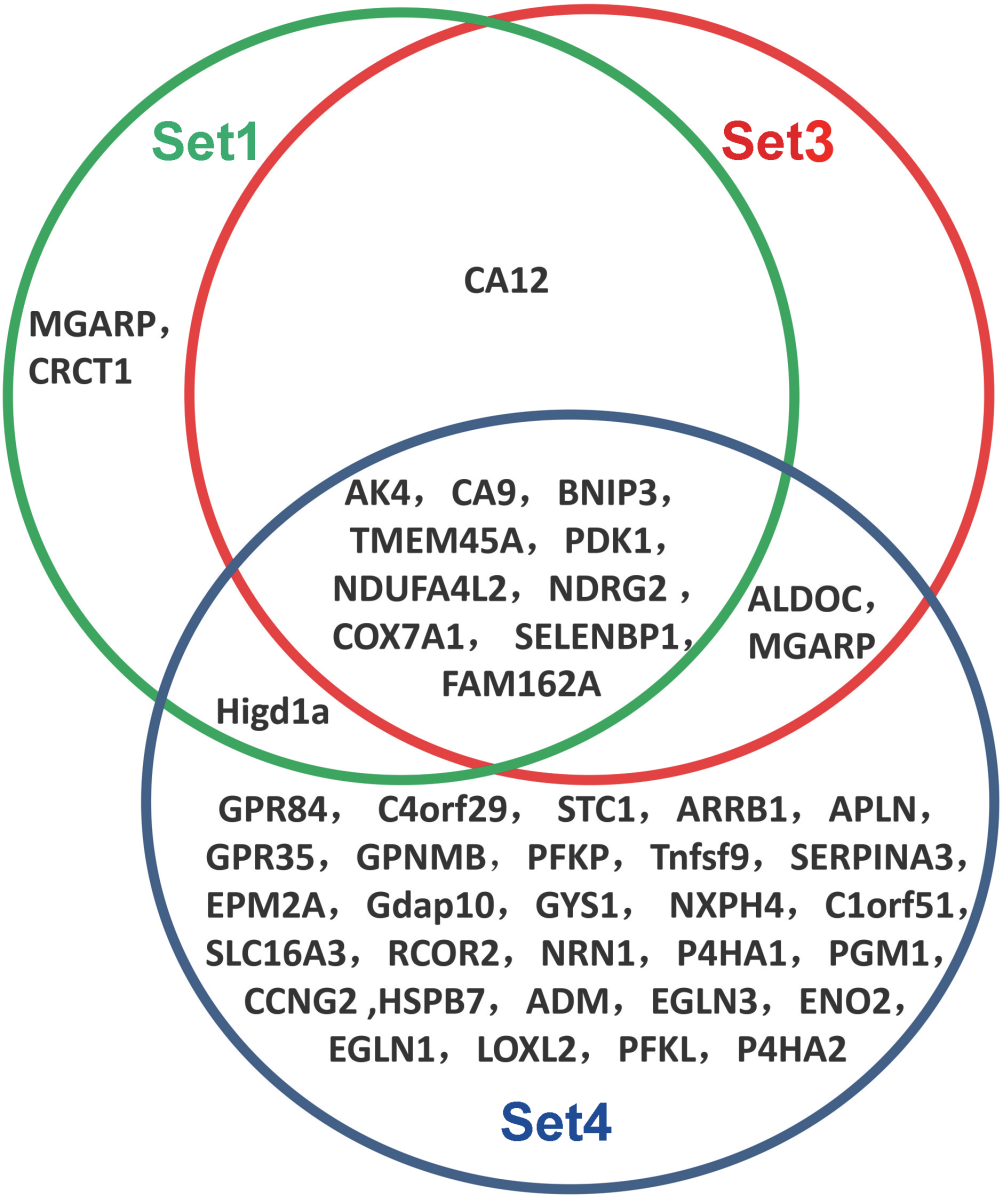
The relationship among differentially expressed genes (FC> 2) in four sets. The relationship of DEGs (FC > 2) among set 1, set 3 and set 4 was further analyzed. Most of DEGs in set 1 and set 3 are same except 3 genes (*CRCT1, ALDOC* and *Higd1 a*). There were one gene (*CA12*) in set 3 and two genes (*MGARP* and *CRCT1*) in set 1 different from set 4. Set 1: μ-g *v.s.* control; Set 3: 1-g *v.s.* control; Set 4: μ-g *v.s.* 2-g.

The relationship of DEGs (FC > 2) among set1, set3 and set 4 was further analyzed (Fig. 2). Most of DEGs in set 1 and set 3 are same except 3 genes (*CRCT1, ALDOC* and *Higd1a*). There were one gene (*CA12*) in set 3 and two genes (*MGARP* and *CRCT1*) in set 1 different from set 4.

### Molecular function and cellular location of DEGs in different sets

In order to further identify interesting new target genes, we used iReport data analysis system to analyze the molecular function and cellular location of DEGs. Molecular function of DEGs (with a 1.5-fold change) in 4 sets was analyzed by iReport data analysis system. More than 20% DEGs in 4 sets belonged to enzyme (Table 2). In set 4, there were 9 and 11 DEGs pertained to transciption regulator and transporter, respectively. In the set 2, the molecular function of 6 DEGs was related to cytokines and transporter, respectively (Table 2). Moreover, several DEGs related to G-protein coupled receptor were presented in all the four sets.

**Table 2:** Functional categories of DEGs in the four sets (FC>1.5).

**Molecular Function**	**Groups**			
	**μ-g *v.s.* control**	**2-g *v.s.* control**	**1-g *v.s.* control**	**μ-g *v.s.* 2g**
cytokine	0	6(*5.50%*)	0	5(*2.98%*)
G-protein coupled receptor	2(*4.26%*)	3(*2.75%*)	1(*2.63%*)	5(*2.98%*)
growth factor	0	1(*0.92%*)	0	2(*1.19%*)
Enzyme	12(*25.53%*)	23(*21.10%*)	8(*21.05%*)	42(*25%*)
Kinase	5(*10.64%*)	5(*4.59%*)	4(*10.53%*)	10(*5.95%*)
microRNA	0	1(*0.92%*)	2(*5.26%*)	4(*2.38%*)
other	23(*48.94%*)	53(*48.62%*)	20(*52.63%*)	70(*41.67%*)
Peptidase	1(*2.13%*)	5(*4.59%*)	0	3(*1.79%*)
phosphatase	1(*2.13%*)	1(*0.92%*)	1(*2.63%*)	4(*2.38%*)
Transcription regulator	1(*2.13%*)	4(*3.67%*)	0	9(*5.36%*)
translation regulator	1(*2.13%*)	0	1(*2.63%*)	0
transmembrane receptor	0	1(*0.92%*)	0	3(*1.79%*)
transporter	1(*2.13%*)	6(*5.50%*)	1(*2.63%*)	11(*6.55%*)

DEGs: differentially expressed genes. This table listed the number and the percentage of DEGs’ functional categories in μ-g *v.s.* control, 2-g *v.s.* control, 1-g *v.s.* control and μ-g *v.s.* 2-g (FC > 1.5).

The percentages of DEGs loacted in cytoplasm ranked the first in all the four sets, and these genes take up 48.9%, 31.2%, 55.3% and 36.3% in set 1, set 2, set 3 and set 4, respectively (Table 2). More than 10% DEGs distributed in plasma membrane in 4 sets. Genes located in extra cellular space in set 2 were more than those in any other three sets (Table 3).

**Table 3:** The percentage of cellular locations in the four sets (FC>1.5).

**Location**	**Groups**			
	**μ-g *v.s.* control**	**2-g *v.s.* control**	**1-g *v.s.* control**	**μ-g *v.s* 2-g**
Cytoplasm	23(*48.94%*)	34(*31.19%*)	21(*55.26%*)	61(*36.31%*)
Extracellular Space	4(*8.51%*)	23(*21.10%*)	2(*5.26%*)	24(*14.29%*)
Nucleus	4(*8.51%*)	13(*11.93%*)	3(*7.89%*)	22(*13.10%*)
Plasma Membrane	7(*14.89%*)	21(*19.27%*)	4(*10.53%*)	33(*19.64%*)
Unknown	9(*19.15%*)	18(*16.51%*)	8(*21.05%*)	28(*16.67%*)

This table listed the number and the percentage of DEGs’ cellular location in μ-g *v.s.* control, 2-g *v.s.* control, 1-g *v.s.* control and μ-g *v.s.* 2-g (FC>1.5).

### Functional annotation clustering of DEGs in different sets

To reduce the burden of associating similar redundant terms and make the biological interpretation more focused, we utilized DAVID funtional clustering to measure relationships among the annotation terms based on the degrees of their co-association genes. We selected the terms with the smallest *P* value and a enrichment score more than 2. Totally, 5 subsets of genes were culsterd based on GO in the three sets (Table 4). The subsets belonged to set 1 and set 4 presented an evident association with the glucose metabolic process (Table 4). These results were enhanced by the subsets in SP-PIR-Keywords and the two groups of genes enriched by KEGG-Pathways (Table 4). Genes of set 2 were clustered into two GO categories, one subset was marked by oxidoreductase activity, and another showed notable location in extracellular region (Table 4). In set 3, the clear clustering genes were not found.

**Table 4:** Functional annotation cluster of DEGs in four sets (FC>1.5).

**Groups**	***P* value**	**Gene symbol**	**Gene group**
GOTERM_BP_FAT			
			
glucose metabolic process	1.03E-07	PDK1, ALDOART1, PFKL, ALDOC, PGM1, ENO2, PGK1	Set 1
glucose metabolic process	7.51E-15	PDK1, ALDOART1, ALDOA, LDHA, PFKL, ALDOC, SLC37A4, EPM2A, PGAM1, PFKP, HK1, PPP1R3C, PYGL, PGM1, ENO2, GYS1, PGK1	Set 4
GOTERM_MF_FAT			
			
oxidoreductase activity, acting on single donors with incorporation of molecular oxygen, incorporation of two atoms of oxygen	1.75E-10	P4HA2, PLOD1, P4HA1, JMJD6, PLOD2, EGLN3, KDM4B, KDM3A, EGLN1, TET2, KDM5B	Set 4
oxidoreductase activity, acting on single donors with incorporation of molecular oxygen, incorporation of two atoms of oxygen	1.53E-09	P4HA2, P4HA1, PLOD2, P4HA3, EGLN3, KDM4B, EGLN1, TET2, KDM5B	Set 2
GOTERM_CC_FAT			
			
extracellular region	8.17E-06	CSF3, BGLAP, TNF, ENPP1, OLR1, MUP1, IL1RN, COL3A1, CCDC80, MCPT8, MMP13, IL10, IGSF10, OLFML3, S100B, ADM, SULF2, AGT, COL6A3, HTRA4, STC1, LOXL2, CSN3, ADAMTS5	Set 2
KEGG_PATHWAY			
			
Glycolysis / Gluconeogenesis	1.94E-05	PFKL, ALDOC, PGM1, ENO2, PGK1	Set 1
Glycolysis / Gluconeogenesis	8.03E-09	ALDOA, LDHA, PFKL, ALDOC, PGM1, PGAM1, ENO2, PFKP, HK1, PGK1	Set 4
SP_PIR_KEYWORDS			
glycolysis	1.10E-06	ALDOART1, PFKL, ALDOC, ENO2, PGK1	Set 1
glycoprotein	7.18E-07	CSF3, SLC5A3, GPR84, CPM, TNF, ENPP1, CD248, PRND, COL3A1, CD53, NRN1, IL10, IGSF10, S1PR3, OLFML3, P4HA2, CLEC4E, P4HA1, PLOD2, ELOVL3, AGT, P4HA3, COL6A3, CLEC4D, LOXL2, GPNMB, OLR1, IL1RN, CCDC80, MCPT8, MMP13, GZMF, GPR35, SNED1, SULF2, STC1, CLEC14A, ADAMTS5	Set 2
signal	5.77E-06	CSF3, CPM, CD248, PRND, COL3A1, NRN1, IL10, IGSF10, OLFML3, P4HA2, P4HA1, PLOD2, AGT, COL6A3, P4HA3, LOXL2, GPNMB, APLN, BGLAP, MUP1, IL1RN, CCDC80, MCPT8, MMP13, GZMF, ADM, SNED1, SULF2, STC1, CSN3, ADAMTS5, CLEC14A	Set 2
dioxygenase	1.62E-09	P4HA2, P4HA1, PLOD2, P4HA3, EGLN3, KDM4B, EGLN1, TET2, KDM5B	Set 2
glycolysis	1.95E-11	ALDOA, ALDOART1, LDHA, PFKL, ALDOC, PGAM1, ENO2, PFKP, HK1, PGK1	Set 4
dioxygenase	1.18E-10	P4HA2, PLOD1, P4HA1, JMJD6, PLOD2, EGLN3, KDM4B, KDM3A, EGLN1, TET2, KDM5B	Set 4

Gene fuctions were annotated based on terms of Gene Ontology, SP_PIR_KEYWARDS and KEGG_PATHWAY. Genes were clustered according to the annotation terms by using DAVID Bioinformatics Resources 6.7 (http://david.abcc.ncifcrf.gov/). The most significantly related terms were selected based on *P* value (Enrichment sore > 2, FDR < 0.05). Set 1: μ-g v.s. control; Set 3: 1-g v.s. control; Set 4: μ-g v.s. 2-g.

**Figure 3:**
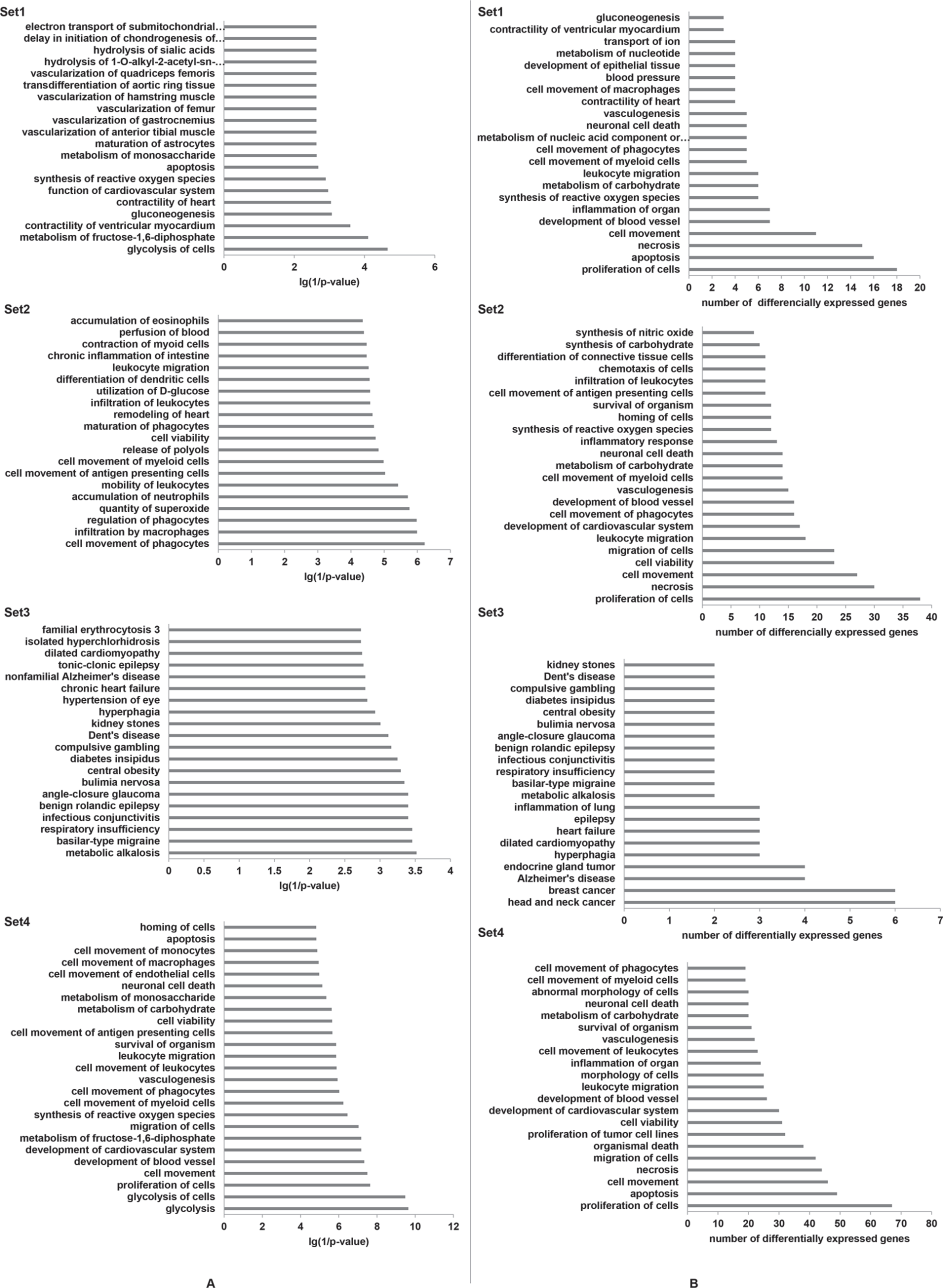
Biological processes associated to differentially expressed genes in four sets. Biological processes were mapped by Ingenuity knowledgebase. Line A shows the the most statistically significant biological processes of set 1, set 2 set 3 and set 4. line B shows the biological processes (*P* < 0.05) involving most differentially expressed genes. Fisher’s exact test was used to calculate the *P* value. Set 1: μ-g *v.s.* control; Set 2: 2-g *v.s.* control, Set 3: 1-g *v.s.* control; Set 4: μ-g *v.s.* 2-g.

Ingenuity iReport can be used to filter, group, and visualize genes by function, biological process, role in pathway or disease. In order to further analyze DEGs associated to biological process, we chose Ingenuity iReport to filter genes statistically significant associated to biological processes. 20 biological processes with the minimum *P* value and with the most number of DEGs were showed in Fig. 3 Glycolysis of cells ranked first in set1 and set 4 because of the minimum *P* value (Fig 3A). The apoptosis process was markedly involved in set1 and set 4 (Fig. 3B). In set 2, multiple biological processes, such as cell viability, cell movement were clustered (Fig. 3A and B). In set 3, the clustering genes are mainly related to disease.

### Verification of DEGs sensetive to distinct apparent gravity levels by qPCR

**Figure 4:**
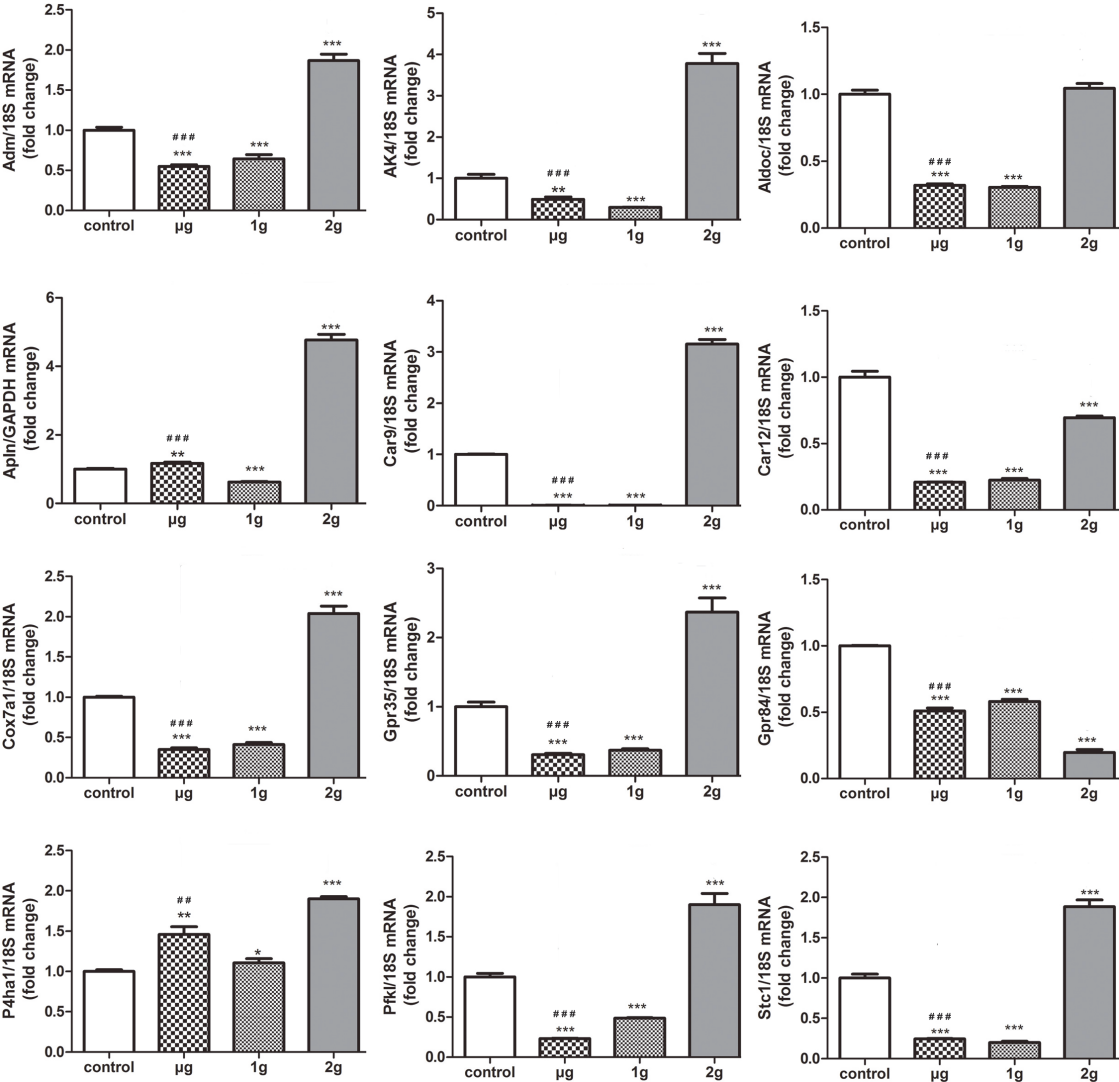
Microarray results were verified using real-time PCR for genes in response to LG-HMF. Total RNA was extracted and qPCR assay was used to further identify for 12 selected genes. The method of relative quantification was used to estimate the relative expression changes of selected gene expression in MLO-Y4 cells exposed to LG-HMF. The changes in selected gene expression, normalized to 18S under LG-HMF were calculated. The difference between μ-g *v.s.* control, μ-g *v.s.* 2- g, and 1- g *v.s.* control was statistically analyzed by one-way ANOVA. μ - g, 1-g, 2 - g *v.s* control group: ***P< 0.001; **P< 0.01; *P< 0.05. μ - g *v.s* 2 - g group: ^###^ *P* < 0.001; ^##^ *P* < 0.01.

In order to verify the corrections of microarray data, we selected 12 DEGs from microarray data and real-time PCR was used to verify the effects of LG-HMF on these gene expression at mRNA levels. The selected DEGs could be classified into 4 subgroups according to functional clustering: enzyme related genes *(CA9, CA12* and *P4H A1*), G-protein coupled receptors (*GPR35* and *GPR84*), peptide hormone (*STC1, ADM and APLN*) and genes related to energy metabolism (*PFKL, AK4, ALDOC* and *COX7a1*). After being normalized by internal control genes, the relative gene expression levels in experimental groups were obtained comparing with those of control groups. And then, the differences in gene expression between μ-g *v.s.* control, 1-g *v.s.* control, and μ-g *v.s.* 2-g were analyzed (Table 5 and Fig. 4). Fold changes of 12 selected genes in μ-g v.s. control, μ-g v.s. 2-g, and 1-g v.s. control by qPCR and microarray analysis were showed in Table 5. The tendency of microarray analysis and PCR results were similar. Except for *GPR84, PH4HA1* and *APLN*, the other 9 genes (*GPR35, PDK1, AK4, ADM, COX7, STC1, ALDOC, CA9* and *CA12*) expression significantly decreased in μ-g *v.s.* control, 1-g *v.s.* control and μ-g *v.s.* 2-g (Fig. 4). The expressions of *GPR 84* obviously decreased in μ-g *v.s.* control and 1-g *v.s.* control but increased in μ-g *v.s.* 2-g. *APLN* expression decreased in μ-g *v.s.* 2-g and 1-g *v.s.* control but slightly increased in μ-g *v.s.* control (Table 5 and Fig. 4).

**Table 5:** Fold change of DEGs tested by qPCR & microarray in μ-g vs. control, μ-g vs. 2- g, and 1- g vs. control by qPCR and microarray assays.

**Gene name**	**μ-g *v.s.* control**		**μ-g *v.s.* 2g**		**1-g *v.s.* control**	
	**PCR**	**Microarray**	**PCR**	**Microarray**	**PCR**	**Microarray**
*GPR35*	0.31	0.52	0.13	0.22	0.37	0.57
*GPR84*	0.51	0.67	2.63	2.38	0.58	0.68
*PFKL*	0.23	0.57	0.12	0.31	0.48	0.62
*AK4*	0.49	0.26	0.13	0.089	0.3	0.37
*ALDOC*	0.32	0.5	0.31	0.37	0.31	0.47
*COX7A1*	0.35	0.36	0.17	0.26	0.41	0.41
*STC1*	0.24	0.6	0.53	0.19	0.2	0.55
*ADM*	0.55	0.66	0.29	0.38	0.64	0.74
*CA9*	0.006	0.28	0.002	0.09	0.007	0.29
*CA12*	0.21	0.45	0.13	0.56	0.22	0.48
*P4HA1*	1.46	—	0.77	0.42	1.11	—
*APLN*	1.16	0.62	0.24	0.28	0.62	0.63

RNA from cells sampled at 48h in LG-HMF and ground controls was evaluated by DNA microarray and by RT-PCR as described in materials and methods. Fold changes of 12 differentially expressed genes in μ-g v.s. control, μ-g v.s. 2-g, and 1-g v.s. control by QPCR and microarray analysis were listed. The fold change between μ-g and 2-g conditions was calculated based on 2^−ΔΔCT^ (Livak) method. 18S or GAPDH was chosen as reference genes. All the changes showed significant differences (*t*-test, n = 3).

### Bio-data mining of the verified genes

**Figure 5:**
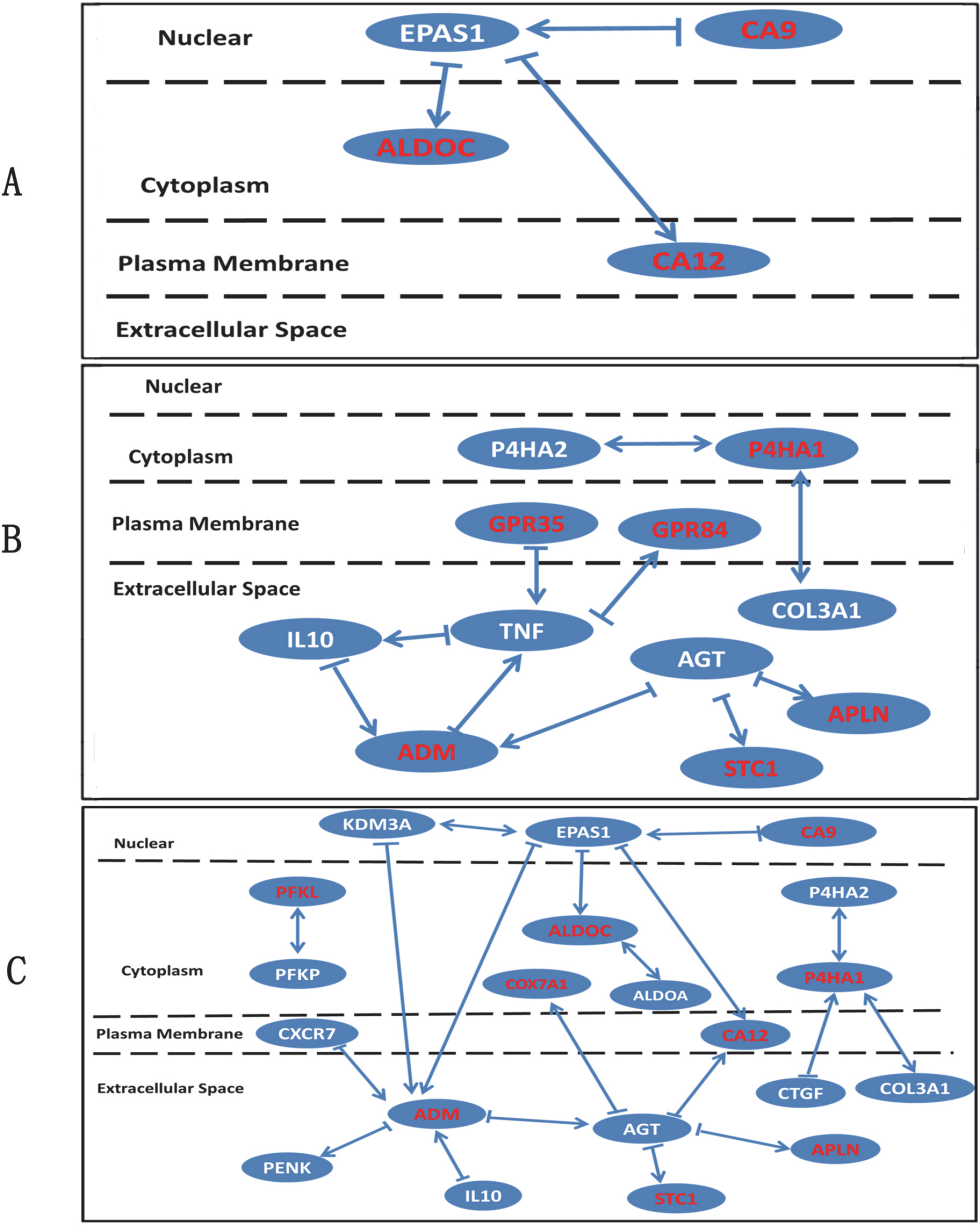
Analysis of interactions among differentially expressed genes in four sets. Interactions of DEGs were mined by iReport (http://www.ingenuity.com/products/ireport) on the basis of Ingenuity knowledge base. The arrow points downstream. The double sided arrow indicated that interaction of the two genes were bi-directional. The red font genes were those verified by PCR, and the write font ones were DEGs tested by microarray. The dashed lines partitioned different regions of osteocyte cell and genes then presented as their cellular locations. (A) μ-g *v.s.* control, (B) 2-g *v.s.* control, (C) μ-g *v.s.* 2-g.

Bio-data mining was performed on the basis of Ingenuity Knowledge Base. Interactions between DEGs were analyzed. We focused on the 12 verified genes and mapped them by the interaction network (Fig. 5). Notably, genes presented much more complex interactive relation in set 2 and set 4 than those in set 1 (Fig. 5A, 5B). However, in set 3 clear interactive relations among 12 verified genes were not found. Interestingly, in view of μ-g *v.s.* 2-g (Fig. 5C), we got several hub genes linking the genes of interest (the red fond genes in Fig. 5). *EPAS1, ADM* and *AGT* (Fig. 5), as well as the *TNF* (Fig. 5B) played as joints between genes. Moreover, according to literature mining of Ingenuity, we mined those genes biological information deeply. The disease processes and pathways associated with DEGs were filtered. Diseases of bone metabolisms were presented with the corresponding set of genes (Table 6). Particularly, *CA9* and *CA12* play role in osteoporosis (Table 6).

**Table 6:** DEGs involved in disease processes in four sets.

**Group**	**Disease term**	**Genes**
μ-g *vs*. control	osteoporosis	CA9, CA12
1-g *vs*. control	osteoporosis	CA9, CA12
	juvenile rheumatoid arthritis	ADM, IL1RN, CCL3L1/CCL3L3, S100A8, TNF
2-g *vs*. control	arthritis	ADM, ENPP1, SLC7A11, IL10, MMP13, CSF3, EGLN1, CLEC4E, IL1RN, CLEC4D, EGLN3, CCL3L1/CCL3L3, S100A8, ADAMTS5, TNF, COL3A1
	abnormal bone density	CA9, CTSK, CA12, Ly6a, CSF3, COL3A1
μ-g *vs*. 2-g	arthritis	ADM, PGK1, CRYAB, CXCL9, SLC7A11, IL10, MMP13, CSF3, CDA, SELENBP1, RASGRF1, EGLN1, CLEC4E, EGLN3, ALDOA, CCL3L1/CCL3L3, TFRC, ENPP2, COL3A1
	dyskinesia	KDM3A, PGK1, PLOD2, CRYAB, CTGF, SLC2A1, NDRG1, ENO2, CA12, AQP1, SERPINA3, P4HA1, USP13, PENK, LDHA, PPARGC1A

Disease processes in which DEGs participated were selected by iReport system (http://www.ingenuity.com/products/ireport) according to ingenuity knowledge base. Fisher’s exact test was used to calculate the statistical significance between the gene and disease term. iReport analysis presented a set of genes involved in one disease process. Results showed in this table were bone-related diseases in which the 12 verified genes involved. (*P* < 0.05)

## Discussion

As a novel technology, the diamagnetic levitation technique has caused more and more attention and has been applied in many fields, such as material sciences, biology, and chemistry. In this study, the effects of diamagnetic levitation on gene expression profiling in osteocytes have been investigated for the first time. Our previous results showed that the cellular morphology and cytoskeleton of osteocytes changed dramatically after cultured in LG-HMF for 2 days, and the expression of genes presented a quite different scene [23]. Based on these results, we further investigate the effects of LG-HMF on the gene expression profiling in osteocytes. The novel and most significant finding is that exposure of osteocytes to LG-HMF (μ-g, 1-g, and 2-g) distinguishes some genes that are sensitive to low gravity, magnet field, and the combined environment. The results are helpful to improve our understandings of how cells sense altered gravity and the mechanisms bone loss induced by weightlessness at a cellular level.

Since a high magnetic field coexists with different gravity levels at all time, four groups were designed in this study, namely 1 g group (normal gravity, 16 T), control group (normal gravity, geomagnetic field), 2 g group (2-fold gravity, 12 T), and μ-g group (hypogravity, 12 T). In order to relatively distinguish the effects of magnetic fields and different apparent gravities, we named four sets as set 1(μ-g *v.s.* control), set 2 (2-g *v.s.* control), set 3 (1-g *v.s.* control) and set 4 (μ-g *v.s*. 2-g). Set 1, set 2, set3 and set 4, respectively, showed the effects of diamagnetic levitation, hypergravity, magnetic field and hypogravity.

By using iReport and DAVID analysis, DEGs in 4 sets were obtained. In set1, set 2 and set 4, the number of down regulated DEGs was much more than that of up regulated DEGs but it was converse in set 3. The results indicate that hypergravity increases most of DEGs expression while hypogravity or magnetic field mainly decreases their expression. Moreover, 10 of DEGs (FC>2) were sensitive to the combined environment (Fig. 2). iReport data analysis also showed that more than 20% DEGs in 4 sets belonged to enzyme, moreover, more than 30% DEGs located in cytoplasm in all the four sets. Manchester reported that space flight obviously affected eleven enzymes in indibidual fibers of soleus and tibilis anterior muscules [41]. The findings suggest that enzyme related genes may be very sensitive to extreme environment.

In order to decrease the similar redundant terms and make the biological interpretation more focused, we used DAVID funtional clustering to detect relationships among the annotation terms. The results showed that DEGs associated with glucose metabolic process or glycolysis were strikingly presented in set 1, set 2 and set 4 groups (Table 4). The results suggest that abnormal gravity may affect osteocytes metabolism. Glycolysis is the universal pathway used by all the organisms to extract energy from glucose. Ramirez *et al.*, reported that when mice were subjected to hind limb suspension, the glycolysis was inhibited, while gluconeogenesis was up regulated in liver [42]. The results given by iReport also confirmed the effects of abnormal gravity on glucose metabolism in osteocytes (Fig. 3). Genes related to the apoptosis, necrosis and cell movement processes were also sorted in set 1, set 2 and set 4 by iReport, but genes in set 3 did not present the similar clustering (Fig. 3). These results indicated that abnormal gravity affected osteocyte functions, such as apoptosis, necrosis and cell movement processes but the high magnetic field did not involve in these processed. Furthermore, all these results suggest that osteocytes might respond the mechanical changes through one or more of these processes.

Totally 12 DEGs were concerned and verified because of their significant changes and their involvements in biological processes. Both of PCR and microarray analysis showed the expression of 12 DEGs (*CA9, CA12, P4HA1, ADM, STC1, APLN, GPR35, PFKL, AK4, ALDOC, COX7A1*) was significantly changed in μ-g *v.s.* control, 1-g *v.s.* control and μ-g *v.s.* 2-g. It suggests that these DEGs are sensitive to both altered gravity and high magnetic field. The carbonic anhydrases (CA) belong to a family of enzymes that catalyze the rapid interconversion of carbon dioxide and water to bicarbonate and protons. *CA9* and *CA12* are two members of carbonic anhydrase family [43–45]. It has been known well that another CA isoform, *CA2*, takes an active part in the bone resorption of osteoclasts by regulating the osteoclastic PH. Moreover, *CA9* and *CA12* had been supposed to act as the Synergy factor of *CA2* [43]. Alteration of CA2 in both flight and suspended animals after readaptation to Earth gravity [46].These evidences taken together prompted that *CA9* and *CA12* in osteocytes might do something crucial in mechano-induced bone remodeling. Prolyl 4-hydroxylase subunit alpha-1 (P4HA1), like *CA9* and *CA12* belongs to enzymes, which plays a key role in collagen synthesis. Decrease in *P4HA1* expression demonstrates that LG-HMF may affect collagen synthesis in osteocytes and ultimately impact bone formation.

Adrenomedullin (ADM) is a peptide hormone that in humans is encoded by the *ADM* gene. ADM was reported to inhibit the osteoclastogenesis [47], while stimulate osteoblast growth and proliferation [48]. Up-regulation of the ADM system occurred early and took part in the adaptative changes occurring during simulated microgravity conditions [49]. The decrease of *ADM* expression in osteocytes under LG-HMF condition suggests *ADM* may be involved in response of osteocytes to the extreme environment. Stanniocalcin-1 (STC1) is a glycoprotein hormone involved in calcium/phosphate (Pi) homeostasis. Filvaroff, *et al*. has reported that STC-1 can affect calcium homeostasis, bone and muscle mass and structure, and angiogenesis through effects on osteoblasts, osteoclasts, myoblasts/myocytes, and endothelial cells [50].The reduction of *STC1* expression in osteocytes under LG-HMF conditions suggests *STC1* may be involved in abnormal bone remodeling process. Apelin (APLN) is a peptide that is encoded by the *APLN* gene, and APLN receptor expression is observed at the surface of osteoblasts, the cell progenitors involved in bone formation [51], meanwhile, lack in APLN increased bone mass in mice osteoblast [52]. Our results showed that *APLN* expression significantly decreased in osteocytes in μ-g *v.s.* 2-g, which indicates that *APLN* may be sensitive to altered gravity.

G-protein coupled receptors, *GPR35* and *GPR84* expression dramatically changed in osteocytes under LG-HMF. GPR35 functions as a receptor for the kynurenine pathway intermediate kynurenic acid, which elicits calcium mobilization and inositol phosphate production [53].GPR84 is highly expressed in the bone marrow, and in splenic T cells and B cells [54] and may link fatty acid metabolism to immunologic regulation [55]. Thesed findings indicate that G-protein coupled receptors may directly or indirectly participate in regulating bone metabolism.

Gluconeogenesis is required for the living organisms to grow at the expense of carbon as energy source other than carbohydrates and capable of synthesizing glucose from simple starting materials [56]. In this study, DAVID funtional clustering showed that several DEGs associated with glucose metabolic process or glycolysis were obviously presented under LG-HMF. PCR and microarray results showed that the expression of energy metabolism related genes, *PFKL* (6-phosphofructokinase, liver type), *AK4* (adenylate kinase 4), *ALDOC* (aldolase C, fructose- bisphosphate) and *COX7A1* (cytochrome c oxidase subunit VIIa), dramatically decreased. These results demonstrate that LG-HMF affect metabolic enzymes in osteocytes. The changes in metabolic enzymes in mice liver or muscle fibers were presented under space flight or simulated microgravity conditions [41, 42].

In order to further investigate the function of DEGs, we analyzed the interaction among DEGs by the interaction network. The results showed that there were several hub genes linking the genes of interest in μ-g *v.s.* 2-g, such as *ADM, P4HA1*. And the gene interaction network also showed that some genes, including *EPAS1* (endothelial PAS domain protein 1), *TNF* (tumor necrosis factor), *AGT* (angiotensinogen) were involved in regulating DEGs. The gene interaction network provides useful clues for further study in future. Disease processes in which DEGs participated were selected by iReport system. Particularly, *CA9* and *CA12* play role in osteoporosis. These results further imply the importance of carbonic anhydrases in bone disease.

In summary, the present study used DNA microarray analysis to provide a new and comprehensive cognition to the effects of LG-HMF on gene expression profiles in osteocyte-like cells, and has selected 12 genes (*CA9, CA12, P4HA1, ADM, STC1, APLN, GPR35, PFKL, AK4, ALDOC, COX7A1*) that may be sensitive to altered gravity or magnetic field. The study shows that LG-HMF affects the expression of several kinds of genes related to enzyme, peptide hormone, G-protein coupled receptors and glucose metabolic process. The identification of mechanosensitive genes will help us to understand the mechanism of bone loss to open a new route for the therapeutic control of bone mass and provide new potential countermeasures.

## Materials and Methods

### Cell culture

MLO-Y4 osteocyte-like cell gifted by Dr. Lynda Bonewald [57] were cultured in α-Modified Eagle’s Medium (α-MEM, Gibco, Paisley, UK) containing 5% fetal bovine serum, 5% calf serum (Gibco, Paisley, UK), 1% benzylpenicillin and 1% streptomycin. MLO-Y4 cells grew on culture flask coated with collagen (rat tail collagen type 1, 0.15 mg/ml, BD, USA). Once reaching 80%–85% confluence, cells were digested by trypsin containing 0.03% EDTA, and seeded onto 96-well plates (9102; Corning Costar,Corning, NY, USA) with a density of 30000 per well, then ten wells were placed into a 35-mm tissue-culture plate (Nunc, Inc., Roskilde, Denmark). And then the plate was delivered to the appropriate (μ-g, 1-g, and 2-g) in the bore of the superconducting magnet by the object holder to continuously culture for 48 hours at at 37°C with 5% CO_2_. The control group was incubated at 37°C with 5% CO_2_ in normal condition.

### Superconducting Magnet with Large Gradient High Magnetic Field

Superconducting magnet with LG-HMF was manufactured by Japan Superconductor Technology, Inc. (JASTEC) according to the specific specifications proposed by authors. Specifications of the superconducting magnet were shown in Table 7. The height of the superconducting magnet is 195 centimeters and a Φ51mm×450mm cylindrical cavity can be used for experiment. The superconducting magnet can generate a magnetic force field (*B* ·dB/dz) of −1370, 0, and 1370 T^2^/m in a 51-mm diameter room temperature (RT) bore, corresponding to three apparent body force levels (*μ*-g, 1-*g*, and 2-*g*) and three magnetic induction intensities (12, 16, and 12 T), respectively. The experimental platform for diamagnetic levitation of biological systems has been further developed based on the superconducting magnet by the authors [39,40].The experimental platform mainly contains four sections: superconducting magnet (JASTEC, Japan) providing large gradient high magnetic gravity environments, temperature control system, object stage, gas control system and observing system. The monitoring device was integrated into the object stage to measure the gravity, temperature, and displacement. The temperature control system includes a water-bath pump and a channel system, and the temperature range for the control system was 37 ± 0.5°C.

**Table 7:** Specifications of superconducting magnet.

**Groups**	**Gravity level**	**Magnetic intensity**	**magnetic force field (B ·dB/dz)**
Diamagnetic levitation	μ-g	12T	−1370 T^2^/m
1-g with LG-HMF	1-*g*	16T	0 T^2^/m
2-g with LG-HMF	2-*g*	12T	1370 T^2^/m
Control	1-*g*	geomagnetic field (30–50μT)	0 T^2^/m

This table listed the specifications of the superconducting magnet, including apparent gravity level, magnetic intensity and magnetic force gradient.

To distinguish gravitational or magnetic field effects, we designed 4 groups in this study, namely, control group (1-*g*, geomagnetic field), diamagnetic levitation group (*μ*-g, 12 T), 1-*g* group (1-*g*, 16 T), and 2-*g* group (2-*g*, 12T). For conveniently describing, we named the four sets as set 1 (μ-g *v.s*. control), set 2 (2-g *v.s*. control), set 3 (1-g *v.s*. control) and set 4 (μ-g *v.s*. 2-g).

### Gene expression profiling by DNA microarray

Total RNA was isolated from MLO-Y4 cells exposed to LG-HMF and controls for 48 h using Trizol method as recommended by the manufacturer’s protocol (Invitrogen, Carlsbad, CA, USA). Gene expressions patterns were examined by Affymetrix Mouse Gene 1.0 ST arrays. Total RNA was extracted by using Trizol reagent (Life technologies, Carlsbad, CA, US) with the standard operating steps given by the manufacturer. The integrity of RNA samples were checked by an Agilent Bioanalyzer 2100 (Agilent technologies, Santa Clara, CA, US), which performed as a RIN number.

Then, qualified total RNA was further purified by RNeasy micro kit (QIAGEN, GmBH, Germany) and RNase-Free DNase Set (QIAGEN, GmBH, Germany). Purified total RNA were amplified, labeled and purified by using Ambion WT Expression Kit (Ambion, US) and GeneChip WT Terminal Labeling Kit (Affymetrix, Santa Clara, CA, US). After that, array hybridization was in process through GeneChip Hybridization, Wash and Stain Kit (Affymetrix, Santa Clara, CA, US) in Hybridization Oven 645 (Affymetrix, Santa Clara, CA, US). Next was washing arrays in the Fluidics Station 450 (Affymetrix, Santa Clara, CA, US). All of these steps above were followed by their special instructions.

In the end, array slides were scanned by GeneChip Scanner 3000 (Affymetrix, Santa Clara, CA, US). At the same, Quantity control of microarray was tested by Command Console Software 3.1 (Affymetrix, Santa Clara, CA, US) with default settings. Raw data was normalized by Robust Multi-Chip Average (RMA) algorithm. All data have been deposited in NCBI’s Gene Expression Omnibus (Qian et al., 2014) and are accessible through the GEO Series accession number GSE62128 (http://www.ncbi.nlm.nih.gov/geo/query/acc.cgi?acc=GSE62128).

### Quantitative Real-Time PCR

RNA extraction was performed all the same as the steps in DNA microarray test. cDNA was obtained by reversing transcription of purified RNA samples by using PrimeScript RT reagent kit (TAKALA, Dalian, China). Gene expression was then examined through quantity real-time PCR (qPCR) with a SYBR Premix Ex Taq Ⅱ kit (Takala, Dalian, China). The PCR cycling procedures were as follow: 95°C 30s, 95°C 10s for denaturation, annealing 20s, 72°C 5s for extension, then plate read on 80°C 2s. 45 cycles were operated from denaturation to plate read.

A relative quantitative analysis method was used to calculate the fold change of differential expression between experimental treatment and control, as well as that between μ-g and 2-g. Messenger RNA-specific oligonucleotide primers were designed by primer premier 5 or NCBI primer pick tools, and their sequences were available in table 8, together with their annealing temperatures.

**Table 8:** *Mus musculus* primers of sensitive genes in MLO-Y4 cells used for quantitative real-time RT- PCR.

**Gene name**	**Primer sequences(5’-3’)**	**Annealing temperature (°C)**	**GenBank Accession no**
Pfkl	F-TGGCTGAGGGATGTGG	60	NM_008826
	R-ATGTGGGTCTGACTGGAAG		
Aldoc	F-TCAACCGCTGCCCACTTC	60	NM_009657
	R-CCATCTCCACTGCCTTCAT		
GPR35	F-ATCACAGGTAAACTCTCAGACACCAACT	62	NM_022320
	R-CTTGAACGCTTCCTGGAACTCT		
GPR84	F-TGCAGCCTTTCTCCGTGGACA	62	NM_030720
	R-TACAGAAGACCGCGCCG		
STC1	F-ATGCTCCAAAACTCAGCAGTGATTC	64.5	NM_009285
	R-CAGGCTTCGGACAAGTCTGT		
Car12	F-CCTATGTTGGTCCTGCTG	56.5	NM_178396
	R-CGTTGTAACCTTGGAACTG		
Cox7a1	F-AAAACCGTGTGGCAGAGAAG	60	NM_009944
	R-CCAGCCCAAGCAGTATAAGC		
P4ha1	F-CTGTTCTGCCGCTACCATGA	60	NM_011030
	R-CCCACTCGTCCTCCTGCTT		
AK4	F-GTGGCTGCGTGAGGCTATTTCTTT	60	NM_009647
	R-CCAGCCTGCCTTAACGTCTTGTGT		
Adm	F-AAGTCGTGGGAAGAGGGA	56	NM_009627
	R-TCTGGCGGTAGCGTTTGA		
Car9	F-ATCACCCAGGCTCAGAACAC	60	NM_139305
	R-TTTCTTCCAAATGGGACAGC		
Apln	F-CCTTGACTGCAGTTTGTGGA	60	NM_013912
	R-GTTCTGGGCTTCACCAGGTA		
GAPDH	F- TGCACCACCAACTGCTTAG	60	XM_001473623
	R- GGATGCAGGGATGATGTTC		
18S rRNA	F-AATCAGGGTTCGATTCCGGA	55	NR0032861
	R-CCAAGATCCAACTACGAGCT		

Primers of 12 DGEs and 18S rRNA were designed based on the sequence of each gene available in GenBank (accession no.) and were synthesized.

### Bioinformatics analysis

We got four groups of comparisons: μ-g *v.s.* control, 2-g *v.s.* control, 1-g *v.s.* control, and μ-g *v.s.* 2-g. iReport online software (Ingenuity Systems, USA) was used to identify the differentially expressed genes (DEGs) of each comparison. The analysis technique for filtering genes was LIMMA [37]. The filtering standard was a fold change cutoff of 1.5, with statistical significance of *P* < 0.05. Cellular locations and molecular functions of genes were mapped to ingenuity knowledgebase through iReport. Bio-data mining processes were also executed by iReport based on ingenuity knowledgebase, which consisted of biological processes, pathways, diseases and interactions. The likelihood of the association between genes and given pathway, biological process, or disease was measured by Fisher’s exact test with the statistical significance *P* < 0.05. DAVID online resource was used to cluster the DEGs. Genes were firstly mapped to three different bio-data categories, Gene ontology, SP-PIR-KEYWORDS and KEGG-PATHWAY. Then genes were clustered according to the corresponding category terms by DAVID.

### Statistical Analysis

Statistically significant differences were determined by Prism statistical software (GraphPad Software Inc., LaJolla, CA, USA). A value of *P* < 0.05 was considered significant in all cases. All data averages or means are accompanied by SDs to indicate the amount of variability in the data.
